# Emotion felt by the listener and expressed by the music: literature review and theoretical perspectives

**DOI:** 10.3389/fpsyg.2013.00837

**Published:** 2013-12-17

**Authors:** Emery Schubert

**Affiliations:** Empirical Musicology Group, School of the Arts and Media, University of New South WalesSydney, NSW, Australia

**Keywords:** expressed and felt emotion in music, emotion locus, contagion, normative dissociation, contrast effect, affect valence, literature review

## Abstract

In his seminal paper, Gabrielsson (2002) distinguishes between emotion felt by the listener, here: “internal locus of emotion” (IL), and the emotion the music is expressing, here: “external locus of emotion” (EL). This paper tabulates 16 comparisons of felt versus expressed emotions in music published in the decade 2003–2012 consisting of 19 studies/experiments and provides some theoretical perspectives. The key findings were that (1) IL rating was frequently rated statistically the same or lower than the corresponding EL rating (e.g., lower felt happiness rating compared to the apparent happiness of the music), and that (2) self-select and preferred music had a smaller gap across the emotion loci than experimenter-selected and disliked music. These key findings were explained by an “inhibited” emotional contagion mechanism, where the otherwise matching felt emotion may have been attenuated by some other factor such as social context. Matching between EL and IL for loved and self-selected pieces was explained by the activation of “contagion” circuits. Physiological arousal, personality and age, as well as musical features (tempo, mode, putative emotions) also influenced perceived and felt emotion distinctions. A variety of data collection formats were identified, but mostly using rating items. In conclusion, a more systematic use of terminology appears desirable. Two broad categories, namely matched and unmatched, are proposed as being sufficient to capture the relationships between EL and IL, instead of four categories as suggested by Gabrielsson.

The distinction between emotion felt by a listener (internal locus of emotion) and emotion expressed by a piece of music (external locus of emotion) has become a firmly established part of research agenda of music psychologists in the last decade. Since the seminal work of Gabrielsson (2002) we have seen evidence that emotion felt in response to music (e.g., “the music makes me feel happy”) is sometimes the same as (“the music is happy”) and sometimes different from (“the music is sad”) the emotion expressed by the music—so called “perceived emotion.” This paper aims to push the debate further by examining the data in the literature published in the decade after Gabrielsson (2002) and explaining why the emotions between the two loci (felt vs. expressed) are sometimes systematically different and sometimes the same. The paper is structured as follows. First, (1) the inclusion criteria and limitations of the review are laid out, (2) some early music psychology research related to emotion locus is presented including an overview of Gabrielsson's paper, followed by (3) a collation of the target literature of this review. Then, (4) the theoretical implications of the literature are discussed, with (5) a proposed reworking of Gabrielsson's locus relationships to accommodate a developed understanding, and to highlight some of the key research questions emerging in the field.

## Inclusion criteria and limitations of this review

### Inclusion criteria

The inclusion criteria for the research tabulated for the review are as follows: (1) studies which made a direct comparison between external locus emotion and internal locus emotion in connection with music listening; (2) studies which use the same response regime for both external locus and internal responses; and (3) studies appearing in peer-reviewed journals in the 10-year period of 2003–2012 (that is, the decade since Gabrielsson's publication). Nineteen studies met all three criteria. The reason for excluding studies that have some connection with emotion locus and music are now explained.

### Limitation 1: expressed and felt emotion data compatibility

When discussion of locus is presented in the research literature on emotion in music, it is most frequently an acknowledgment that a locus distinction exists, but that the study limits the investigation to one locus or the other (internal or external), without comparing both. Sometimes the data in each locus are not directly comparable (e.g., a rating of felt emotion, but a categorical, *a priori* label for the emotion expressed by the stimulus), as will be discussed below. Such studies were not included in the tabulated literature of this review.

Thus, studies that could allow comparison of locus by rating of expressed emotion *a priori* (e.g., by a panel of experts, as in numerous mood induction studies), or comparison of physiological measures with one locus or the other (e.g., Krumhansl, 1997; Grewe et al., 2007; Nagel et al., 2007; for a critical review, see Konečni, 2008; Grewe et al., 2009, pp. 263–265) were not included. The bulk of the *a priori* rated expressed locus data are found in the well-established music mood induction literature^1^.

### Limitation 2: terminology to describe the felt/expressed distinction

As discussed below, the terminology to describe locus of emotion is varied, making online keyword searching alone inappropriate for locating articles that fulfill the inclusion criteria^2^. Instead, a number of sources and databases were consulted—Google Scholar, PsycINFO, Scopus, and Web of Knowledge, as well as papers that cited Gabrielsson's (2002) paper.

### Limitation 3: emotion locus research in non-music research

Although an interest in comparing locus in other fields of research can be found—for example, in social reception (e.g., Jakobs et al., 2001; Bombari et al., 2013), cross-modal (e.g., Calder et al., 2000), film (e.g., Matsumoto and Kupperbusch, 2001; Wang and Cheong, 2006; Werner et al., 2007), literature (e.g., Oatley, 1995; Green, 2004; Miall, 2011), business (e.g., Pugh, 2001), and facial expression of emotion (e.g., Dimberg et al., 2000; Barthomeuf et al., 2012; Sato et al., 2013) research—it appears that most interest in the direct comparison of internal with external loci of emotion via empirical means is rooted in music perception research, making the transference of findings from other sub-disciplines limited at this point in time. However, some relevant issues from non-music studies are mentioned in this review.

### Limitation 4: philosophical issues and mood induction

The review is limited to empirical data from music psychology research in which data from each emotion locus (felt by listener and expressed by music) are gathered and compared. It should be noted that music psychology has been influenced by ideas about emotion locus that were primarily in the realm of philosophy and aesthetics. It is those scholars who introduced terms of emotivism and cognitivism, often quoted in the music psychology literature (Baumgartner, 1992; Goldman, 1995; Scherer and Zentner, 2001; Rickard, 2004; Schubert, 2007a; Konečni, 2008; Konečni et al., 2008; Roy et al., 2008, 2009; Coutinho and Cangelosi, 2009; Lundqvist et al., 2009; Garrido and Schubert, 2011b; Hoeckner et al., 2011; Panagiotidi and Samartzi, 2012). However, I do not focus on these philosophical writings not just because they fall outside the “empirical research” gamut, but because they generally have a different focus from that of music psychology: where music psychologists seek to understand the nature and relationship between emotion loci in music, philosophers of aesthetics are frequently concerned with identifying the *value* of music that evokes emotion (the emotivist perspective) vs. expresses emotion (the cognitivist perspective). For example, Scruton (1983) writes, “To describe a piece of music as expressive of melancholy is to give reason for listening to it; to describe it as arousing of evoking melancholy is to give a reason for avoiding it” (p. 49), and (Kivy, 1989) “one substantial group of listeners who report that sad music makes them sad are simply (and understandably) mistaken” (p. 163). This *value* perspective has relevance in music psychology, particularly when value is operationalized as a variable such as liking (some empirical research has provided evidence supporting the spirit of the just cited statements—see, e.g., Konečni et al., 2008; Hunter et al., 2010; Vuoskoski and Eerola, 2012); however, the literature review is limited to *understanding the relationships* between emotion loci from the perspective of listener self-reports (rather than judging the value of the music). Outside such circumstances, the more purely philosophical research will be used to inform the reviewed material, rather than be part of it. Hence, a limitation of the present review will be to examine exclusively music-psychology literature (for excellent aesthetician accounts, see Radford, 1989; Kivy, 1990, 1999, 2002; Davies, 1994, 2003; Robinson, 1994).

### Limitation 5: within-locus distinctions

On a related matter, this review will not attempt to separate “within emotion locus” distinctions, such as the internal locus distinction between inducing mood vs. feeling an emotion (e.g., Weld, 1912; Diener et al., 1995; Lychner, 1998; Gray et al., 2001; Sloboda and Juslin, 2010). Another within-internal-locus distinction is between “truly,” internally felt emotion vs. the emotion one displays in an intrapersonal, social, or work setting. This kind of distinction is covered in non-music research on emotional regulation, which includes protective buffering (Langer et al., 2007; Manne et al., 2007), display rules (Ekman, 1972; Matsumoto, 1990), and emotional dissonance and emotional labor (Bono and Vey, 2005; Mann, 2005; Bakker and Heuven, 2006). Such distinctions within internal locus are not reported here because they have not been cited in empirical music perception investigations that meet the inclusion criteria, and do not at first *seem* to be of relevance because one would imagine that knowing how to behave in front of a piece of music is not relevant in the way that knowing how to behave in front of other people is relevant. That is, it seems unlikely that we would need to distinguish between how we actually feel and the felt emotion that we display (for example, through facial, bodily or written/typed response) in the study of emotion in music. Although this internal locus distinction is not covered in the empirical data of the literature reviewed, it will be relevant in future research (for a discussion in non-music contexts, see Gross et al., 2000).

Within-external locus distinctions, such as whether music is *portraying* emotion, *expressing* emotion, *trying* to portray/express emotion, or “is” emotional (the music *is* sad), are also not covered in this review, again because empirical data investigating these distinctions are rare and mostly concerned with semantics. Instead, in this review, the terminology used to discriminate external and internal locus (rather than its semantic/linguistic utility) is reported (see subsection “Locus Terminology”, below), with the exception of one study that is included (Van Zijl and Sloboda, 2011) because it raises the matter of the “performer” locus of emotion, which is external from the listener perspective unless the listener *is* the performer. Again, future empirical studies will be needed to examine the various external locus possibilities, specifically the emotion that the composer(s), performer(s) or other (perhaps imaginary) listener(s) are thought to be experiencing (according to the perceiver), and whether a further distinction between these other people and the music should be made when considering external locus. For example, some recent research has considered emotion ratings that others would make about a piece of music as a way of managing possible bias in external locus response. That is, instead of being asked what emotion they believe the music expresses, a participant is asked, “How would normal people feel when listening to this musical stimulus?” (Kawakami et al., 2013).

## Previous psychological accounts leading up to gabrielsson's publication

Pre-review period (before 2003) accounts by music psychologists demonstrating an awareness of the distinction between felt and expressed emotion are frequent (e.g., Weld, 1912; Valentine, 1962, p. 10; Swanwick, 1975; Payne, 1980; Thayer, 1986; Gaver and Mandler, 1987; Frances, 1988, p. 243; Sloboda, 1992; Scherer and Zentner, 2001; for a discussion of key pre-review period studies, of course see Gabrielsson (2002) but in particular pp. 124–127 and pp. 132–133), but almost none of these explicitly compare locus responses empirically (exceptions among which are Lee, 1932; Collins, 1989; Zentner, 2000).

From a historical point of view, and from a theory-building perspective, it is worth dwelling for a moment on the pioneering study by Lee (1932). A questionnaire was developed to investigate responses to music made by music lovers, to tease out the role of emotion (among other things) reported by participants, and to identify any skepticism about whether music was capable of stimulating human expression and emotion. Over 100 responses were collected, spanning a period of more than 25 years. One part of the analysis was to classify “listeners” who had aesthetic interest in music and “hearers” who were more focused on human-emotional interests in music listening. Lee asked explicitly about locus, but upon analysis she noticed that participants treated the three options of the questions as “one of three” rather than two of three (A or B—internal locus, and then C—external locus):

- Does music put you into emotional conditions (or moods) different from the one you happen to be in? or
- Does it merely intensify already existing moods?
- Do you merely recognize, without participating, that music represents varieties of human emotion and mood? (p. 202)

Lee's openness and regret are explicit as she acknowledges:

What I wanted to know was whether the Answerer merely recognised that a given piece of music had (i.e., might be described as having) a given emotional character, e.g., was sad or cheerful, or whether hearing that piece of music made him feel sad or cheerful when he had not been so before? This fairly simple query, clearly worded as “do you merely recognize without participation that music *represents* varieties of human emotion and mood?” was the real subject under examination and ought therefore to have been put first and foremost. Instead of that, and from a mistaken hope of additional clearness, it was put after the queries (intended to be supplementary to it) whether the Answerer's already existing mood could be altered or whether that mood was merely intensified by the emotion which was not merely recognized as characterizing the music, but actually participated in when hearing that music. As a result of this strategical blunder, the majority of Answerers did not notice the main question of Participation versus Recognition. (p. 203, italics in original)

Nevertheless, Lee identified participants who explicitly reported the link between felt and expressed emotion; for example, two participants, Bob and Lewis, respectively, wrote:

“Music generally substitutes new moods and emotions: if the emotion is tragic or tender, it seems that my mood becomes tragic or tender.” (This is recognition producing a sort of sympathetic imitation.)

[…]

“It is not that the music expressed one's own feelings, but that the feelings or mood which the music expresses awaken these very feelings in oneself. Music never intensifies existing feelings, it either awakens feelings which I haven't got or merely represents them.” (p. 204, Lee's annotation of Bob's comment is shown in the parenthetical)

Hence, Lee's work appears to be the first explicit attempt in English language music psychology research to collect empirical data on the distinction between locus of emotion, a pioneering effort that has received regrettably little attention in subsequent research. Her explanation of “sympathetic imitation” will be highly relevant in the theory development of this review. Aside from the data and analysis reported by Lee, it was not until 2002 when Gabrielsson acknowledged in explicit terms the possible relationships between loci (Gabrielsson is also an important researcher, as was Lee, in compiling a large body of data on individuals' self-reported aesthetic/emotional reactions to music: Gabrielsson, 2011).

Gabrielsson's (2002) publication *was* noticed and paved the way for a new era of research, by laying out the possible relationships between felt and expressed emotions. Gabrielsson provided a cautious, broad definition of emotion, which is used as a starting point in the present review: “Not to get trapped in … terminological confusion already from the beginning, I will use ‘emotion’ and ‘feeling’ in a generic and broad-minded sense, often involving cognitive components; “mood” and “affect” will be used when employed by authors referred to in the text” (p. 123–124). He then presented a detailed argument explaining that participants can only logically make distinctions between felt and perceived emotion in music through verbal report, as distinct from, say, physiological measurement.

Gabrielsson discusses some of the reasons for the absence of direct emotion locus comparisons in music perception. For example, he recounts the belief that there were different listener types, some who focused on the music, and others who focused on their own feelings when listening to music (Lee, discussed above, provides a case in point). Music was seen by many researchers and lay people as “an object for perception and reflection” and the emotional response as a listener's reaction. As a result, Gabrielsson concluded that the distinction between emotion loci in music “is not always clearly observed, neither in everyday conversation about emotions, nor in scientific papers” (Gabrielsson, 2002, p. 124). Another reason for the neglect is the influence of aestheticians upon music psychologists, as mentioned in Limitation 4, above, where the interest was in whether music that expresses emotion should be more valued than music that evokes emotion in the listener. This perspective suggests that each locus has a distinct aesthetic function, providing one reason why researchers have been distracted from the psychological *relationships* between the emotion loci.

According to Gabrielsson, in broad terms, felt and expressed emotions could be related through a “positive” relationship (felt emotion is the same as expressed emotion—feeling sad when hearing sad music), or it could be “negative” (i.e., opposite, e.g., feeling angry when hearing happy music). Furthermore, felt and expressed emotions could exhibit “no systematic relationship” (e.g., feeling various emotions when hearing calm music), or have no relationship at all (such as feeling no emotion, or identifying no emotion in the music). Gabrielsson traces back the presence of positive relationships to the ancient Greeks where music was thought to be able to directly affect the listener, in a manner similar to more contemporary research on mood management and regulation theory (Knobloch and Zillmann, 2002; Saarikallio and Erkkilä, 2007; Wilhelm et al., 2013), through a process that would later be called contagion by Juslin and Västfjäll (2008). That is, contagion explains how music can express an emotion that then “infects” its listener.

Opposite relationships, Gabrielsson explains, and is confirmed by subsequent research, are more idiosyncratic (e.g., when an event of a contrary emotion happens in one's life that becomes associated with music heard at the same time), or a sad piece of music makes the listener happy because it is relaxing, cathartic and pleasurable (Schubert, 1996; Matsumoto, 2002; Huron, 2011; Vuoskoski and Eerola, 2012; Vuoskoski et al., 2012; Schubert, 2013), or the listener, perhaps in attempting to improve mood, actually makes their mood worse, perhaps due to some complicating circumstances such as a mood disorder (Garrido and Schubert, 2011b, 2013).

In the case of no systematic relationship, the listener might not be affected by the music at all, but be able to observe the music as expressing some emotion—Gabrielsson characterizes this with the “analytic listener”—or that different emotions are evoked in the listener at different occasions—the “zero correlation” relationship. The final category, of “no relationship at all,” is characterized by the absence or unreliable presence of emotions in one or both loci, such as the internal locus (human) emotions as identified by Scherer and Zentner (2001) that music cannot express with reasonable agreement, which is likely to include gratitude, fascination, disgust, jealousy, safety, warmth and humility. Gabrielsson's relationships are not intended as clear-cut, and polychotomous: The differentiation between internal and external locus can be blurred, and the experience felt vs. the perceived emotion in the music may not even be distinguishable or meaningful to some. Gabrielsson writes, “We may think of them as opposite extremes on a continuum from ‘pure’ emotion-free perception at the one end to intense emotional reaction at the other end. Rather than being at any of these extremes, in most situations listeners are probably somewhere along this continuum, depending on many circumstances” (p. 124).

In the years following Gabrielsson's influential paper, research on explicitly collected self-report felt and expressed emotion in music grew, and 16 peer-reviewed publications that met the inclusion criteria were located. Some of the publications reported more than one study that was concerned with emotion locus and music, and two reported locus data from previously published sources (Schubert, 2007a; Ilie and Thompson, 2011), bringing the total number of included, unique data sets (studies) comparing emotion loci in music to 19. Fifteen of the studies were experimental (Schubert, 2007a is not added to this count because it is a reanalysis of Schubert, 2007b), three were survey based (no music played), and one was a qualitative study (Van Zijl and Sloboda, 2011).

## Review of the contemporary literature: methodological issues

A summary of the included studies is tabulated in Supplementary Table 1. The reviewed papers are discussed according to terminologies and methodologies, followed by key results.

### Locus terminology

The labels used to denote internal and external locus varied considerably across the tabulated studies. Naming the locus *variable* produced several alternatives: instruction condition (Vieillard et al., 2008: “instructed to report own emotion or instructed to describe the music”), “response” (Hunter et al., 2010), “mode” (Dibben, 2004), “modality” (Zentner et al., 2008), “type of rating” (Ali and Peynircioǧlu, 2010), “point of view” (Kallinen and Ravaja, 2006), and “locus” (Schubert, 2007b).

The labeling of each of the two levels of the variable demonstrates a rich variety of ways of understanding the phenomena in question. Lee's pairing (“participate” for internal locus and “recognize” for external) is absent in all the contemporary literature reviewed, although Vieillard et al. (2008) use the term “recognize” in their recognized-experienced level labels. Collins (1989)—not part of the tabulated review—provides a rather detailed distinction between internal and external loci: “own emotional response—emotional content of the music” and “describe music—describe human emotion,” as do Kallinen and Ravaja in asking their participants to respond to the music from two different “points of view”:

Participants were first asked to evaluate the emotions the music aroused in them during listening (i.e., emotion felt; “How did you feel when you listened [to] the music?”), and second, evaluate the emotional quality of the music regardless of the experiences it aroused in them (i.e., emotion perceived; “What is the [more objective] emotional nature of music, regardless of your personal reactions to it?”). (Kallinen and Ravaja, 2006, p. 200)

Other variants were expressed-felt (Dibben, 2004); perceiving-feeling (Hunter et al., 2010); expressed-induced (Zentner et al., 2008); perception-induction; perceive-experience (Juslin and Laukka, 2004); conveyed-elicited (Ali and Peynircioǧlu, 2010); and participant believed the composer was intending to convey—felt in response to the musical excerpt (Salimpoor et al., 2009). Van Zijl and Sloboda (2011) refer to musical emotion—own emotion. This study is interesting from the perspective that it tracks the responses of the listeners who are also the performers of the music. Such an approach may be incorporated into the “locus” nomenclature used by Schubert, by extending the meaning of external locus beyond the “perspective” of the listener. That is, if the listener is asked to judge the emotion that the performer is experiencing while playing, or the emotion the composer was experiencing while composing, or even the emotion experienced by any other listener (or an imagined agent), the locus would still be external, but focussed on another person, rather than on the music (a detailed investigation of the appreciation of the performer/composer emotion is beyond the scope of this review, but see, for example, Juslin, 2000; Juslin et al., 2001; Kreutz et al., 2008b).

In summary, no single pair of designations for each locus level was used consistently across the studies. This reflects a richness in the descriptions, subtle distinctions, but also lack of systematic classification. The recommendation of this review is to label two levels of the variable as deemed appropriate in each study: felt, induced (although this may be related to mood according to some researchers), evoked, internal locus vs. expressed, portrayed, “in the music,” and external locus. “Perceived” is frequently used to describe external locus, however, the term could be confused because a participant can perceive many things, including their own feelings in some cases (Konečni, 2008). Context and grammar is important here: to make the locus distinction clear from the *listener's* perspective, and when the listener is the subject of the sentence, she or he feels an emotion, but *perceives* it (rather than expresses it) in the music—“express” does not designate external locus when the listener is the subject of the sentence (“the listener expresses happiness”). When the *music* is the subject of the sentence, *expressed* emotion is the external locus designation (obviously not “perceived,” because music is not an agent that can literally perceive).

Using the term “locus” to describe the variable frees up labels that are commonly used to describe other variables, such as type and mode. “Perspective” is another possible term that could be used to describe the variable but was not cited with any regularity in the tabulated literature.

Table 1 attempts to organize all of the terms used to describe each of the two levels of loci located in the literature. It should be understood, then, that the unambiguous use of the terminology depends on the explicit and implied grammatical subject (that is, if stated from the perspective of the listener or of the music). Any of the internal-external pairings are satisfactory but need to have the subject (music or listener) explicitly stated or made clear. The term “communicates” is listed under internal locus from the music perspective because it suggests a context: “the music communicates to me.” But since the term “communication” refers to the *transmission* of information from one source to another, it may lead to some ambiguity (e.g., “the music is communicating an emotion”—external locus?).

**Table 1:** **Terminology of emotion locus levels by grammatical subject (perspective)**.

**Locus**	**Listener perspective**	**Music perspective**
Internal	Felt, experienced, self, own, reactivity	Conveys, induces, evokes, arouses, elicits, (communicates)
External	Perceived, recognized, sensed, noticed	Expresses, portrays, depicts, conveys, sounds, describes, has character, “is”

### Participants

All included experimental studies in this review had over 25 participants in each experiment, with the Konečni et al. (2008) study having 144 participants. The two questionnaire-based studies had 262 (Zentner et al., 2008) and 141 (Juslin and Laukka, 2004) participants. The open-ended study of the musicians' perspectives used 8 performers covering a variety of musical instruments (Van Zijl and Sloboda, 2011). Participants had a range of ages across studies, with one study examining results of older participants separately (Schubert, 2007b; Experiment 3). A wide range of musical experiences were reported, though none of the studies treated musical experience (e.g., high vs. low) as an experimental variable.

### Types of music used

The bulk of music used in the studies tabulated comes from the common practice period (CPP) of Western art music, which is sometimes referred to as “classical” music. Twelve publications using experimenter-selected pieces used exclusively such music. Classical music, and in particular music from the romantic art music period, is considered particularly good for expressing and evoking emotions (Romantic(ism), 2013).

Since studies by Panksepp (1995), Blood and Zatorre (2001), and Rickard (2004), it has become evident that another effective way to evoke strong emotional responses is to use music that the participant, rather than the experimenter, selects. The Rickard study comes near the halfway mark of the review sample chronology, and it is after this date that we start to see self-selected pieces being used for locus of emotion in music studies. The locus studies reviewed first commenced using self-selected music from 2007 (Schubert, 2007a). Salimpoor et al. (2009) directly compared participant-selected and experimenter-selected pieces in their study.

Some research deliberately selected music that is unfamiliar. In the two experiments investigating emotion locus by Dibben (2004), she verified that the music was not familiar to any of the participants. Hunter et al. (2010) also selected unfamiliar music by Bach—eight selections—which were manipulated by tempo and mode to generate the 30 stimuli presented via MIDI playback. Thus, music of the common practice period presented a great range of choices for a Western-enculturated participant. Vieillard et al. (2008) is the only study in the tabulated literature where specially composed music was created for the purpose of exerting a high degree of control over the stimuli. This was balanced with some more familiar film music excerpts, used at the beginning of the procedure. The study by Van Zijl and Sloboda (2011) used self-selected pieces, but these were for the purpose of learning to play—so that participants would be getting to know their stimuli in a highly intimate way.

Two publications reviewed included no explicit musical stimuli (Juslin and Laukka, 2004; Zentner et al., 2008) because data about music in general were collected via questionnaire. The questionnaires provided critical data directly addressing the question of locus. In this respect, they fulfilled the inclusion criteria of this literature review because the participants were asked to think about music in general, or a favorite piece (see also Evans and Schubert, 2008) or genre of music. In addition, Juslin and Laukka (2004) was one of the first published studies (along with Dibben) to directly address the question of locus in the post-Gabrielsson period.

When musical examples were selected, they were usually chosen on the basis of which emotion they would express or evoke, with the intention commonly being to produce a range of emotions, whether based on the theory of basic emotions—such as “joy, sadness, anger, and fear” (Kallinen and Ravaja, 2006) or a sample from each quadrant of an emotion space (e.g., Dibben, 2004; see also Collins, 1989, though not tabulated for the present review). For the experimental studies, the typical length of the excerpts used ranged from 1 to 3 minutes. Ali and Peynircioǧlu (2010) used stimuli thought to be unfamiliar, each lasting approximately 20 seconds, but in one condition, participants were familiarized by listening to an excerpt five times in succession. In one of the two Ilie and Thompson (2006) studies the stimuli used had an average duration of 6 seconds.

### Design and procedures

Many of the tabulated studies ask the participant to perform tasks in a laboratory setting and often in groups. However, the group-listening laboratory setting does not necessarily reflect the typical day-to-day listening experience or environment (for a discussion of the matter of naturalistic vs. experimental research, see Mitchell, 2012). Since the emerging ubiquity of personal, private online computer facilities, tablets and mobile phones (Krause and Hargreaves, 2012), it has become possible to collect sophisticated data outside the laboratory and in a private, individual environment (e.g., see Reid et al., 2009). Such technological advances will allow locus data to be collected in a variety of settings. For example, one of the studies outside the review epoch (Schubert, 2013) used an online survey to collect locus data, requesting participants to use YouTube or some other online streaming resource to listen to their self-selected pieces, and provide information about the piece and the URL (that is, the participants pasted the link they used to access the selected music to allow later inspection by the researcher). The survey could be completed in private. Further developments of this approach will be able to better ascertain the reliability and validity of collecting data in such a way, as compared to group settings with a researcher present and with predetermined ordering of stimulus presentation.

Juslin and Laukka (2004) used a survey with no musical stimuli to gather data on a range of issues regarding musical experiences and beliefs, including emotion perception and emotion induction. The survey included questions about the utility of certain words for describing emotion felt and emotion expressed. This kind of task is easy to administer and produces a rich source of data about emotion locus. Zentner et al. (2008) also used a survey-based approach with a direct rating for each of the two loci for a series of 146 “feeling terms” for a variety of musical styles. Participants first rated the terms as emotions felt for a selected (favorite) musical style, and then again for the emotion perceived in that style.

Indeed, one concern in the literature is the timing of the locus tasks. Should they both be completed immediately after hearing the musical stimulation, one after the other, or should only one be completed (internal only *or* external only) in case one rating influences the rating of the other locus? Completing the two rating items in immediate sequence has the advantage of requiring only a single pass of the music stimulus to obtain both ratings, but has a drawback if the participant (consciously or otherwise) responds to the second rating item under the influence of the first, through what is known as a “contrast” effect (Cacioppo and Gardner, 1999; Schwarz and Strack, 1999; Cheng, 2004), where the second response is made relative to the first, while the first rating item response is (probably) not. For example, a high rating of felt emotion might be exaggerated (rated even higher) if the external locus rating is made immediately before but is also rated as high.

Kallinen and Ravaja (2006) asked participants to rate felt emotion first so as not to dilute the felt emotion caused by the delay of rating expressed emotion first. This ordering was used in several other tabulated experiments (e.g., Dibben, 2004; Schubert, 2007b, Study 2 and 3; Evans and Schubert, 2008). However, Konečni et al. (2008) argued that by rating external locus first (i.e., the emotion in the music), the participant will be more cognizant in distinguishing their own internal locus response and not confuse it with the expressed emotion. They resolved the matter by counterbalancing—half the participants made their internal locus rating first, while the other half made their external locus rating first. Dibben (2004; Experiment 2), Vieillard et al. (2008), and Ali and Peynircioǧlu (2010) each had one internal locus group and another external locus group, each group completing questions for one locus only as a between-subjects design. Dibben (2004) concluded that when participants make a judgment in one locus alone, they do not differentiate between emotion and locus as well as they do when loci are presented together (p. 111), suggesting that some “contrasting” of rating items may be methodologically beneficial, as small differences are amplified. Schubert (2007b) findings were different but led to a similar conclusion: a contrast effect was not observed, and in fact, locus tasks performed together produced some “interference” (Gabrielsson, 2002, p. 127; Dibben, 2004, p. 95) leading to a more blended response, compared to performing one locus task at a time. But the same *trend* in response was noted when compared to the “locus-separate” condition (recording response to one locus only, and then the other on a second hearing of the stimulus), leading Schubert to conclude that responding to both loci in sequence at the same time had efficiency (almost halving experiment time, or “doubling” the data pool, and as a result increasing statistical power), perhaps compensating for the possible disadvantages. The recommendation of this review, then, is that it is more efficient to collect both locus ratings together, but to counterbalance the order of loci questions, as did Konečni et al. (2008) when possible. Most of the tabulated studies, when presenting the loci responses together, requested internal locus rating first. It is not always clear in the literature whether participants could change their answers, though it seems that no effort was made to prevent or withhold the option of checking or changing responses. Explicit investigation of this counterbalancing is recommended.

### Response format

Fifteen of the nineteen tabulated studies were defined here as “experimental,” and for several of those, as well as the three survey studies (see Supplementary Table 1, rows 3, 10, and 12), a wide range of emotions were presented as entities to be rated by the participant after listening to an extract of music, once for internal and once for external locus. This subsection therefore examines these item-rating response formats according to (1) the emotions rated, (2) the number of steps available on each rating item, (3) the number of items rated in each locus for each piece of music, and (4) unipolar versus bipolar item labeling issues.

#### Emotions rated

The most frequently used pole labels (whether rating item was bipolar or unipolar) were happy (including happiness and very-happy) and sad (including sadness and very sad), both used in at least^3^ eight studies (Supplementary Table 1, rows 1, 2, 4, 9, 11, 13, 14, and 16. Note: All subsequent parenthetical references to row numbers in this subsection refer to this table, with focus on the Measure column). In (at least) five studies an item for rating happiness and another for rating sadness were presented (rows 1, 2, 11, 14, and 16), but on three occasions terms related to happy and sad were presented together at the opposite poles of a single, bipolar rating item (rows 4, 9, and 13), such as very happy to very sad, as used by Konečni et al. (2008, row 9). Wording related to “arousal/aroused” was located in four studies (rows 4, 5, 13, and 18—one of which had poles labeled “energetic/peppy” and “bored/vegetated:” Kallinen and Ravaja, 2006, row 4). If we group together the remaining labels according to similarity of meanings, the next most frequently used rating item labels in the tabulated literature are five occurrences related to anger (“angry”, “fear”, and “scary”^4^: rows 1, 2, 4, 11, and 16) and four related to calm (“calmness”, “relax”, and “peacefulness”: rows 4, 11, 16, and 18).

Typically, the rating items were combined and manipulated to create dependent variables for statistical analysis and hypothesis testing. This transformation process is summarized in the Measure column at “DV” for each of the relevant tabulated studies in Supplementary Table 1. The most frequently used labels for dependent variables were related to “valence” (positive, negative, and/or valence: rows 1, 2, 4, and 18) and “arousal” (rows 4, 8, 13, and 18), although untransformed rating items also received these labels in some studies (e.g., row 5). Thus, the emotion constructs of valence and arousal have endured as methods of operationalizing emotion locus response.

#### Number of steps per rating item

When making a response via a rating item, participants are given a number of graded steps (points) along a continuum from which they are to make a single selection. The range of the available steps per rating item across the tabulated studies was from 4 to 13, with 5-point rating items used most frequent (seven studies, rows 1, 2, 4, 10, 14, 16, and 18). Researchers have had to balance the coarseness of completing rating items with fewer points with the greater difficulty for the participant in making a selection when a larger number of steps are offered (Alwin, 1997; Viswanathan et al., 2004; Dawes, 2008). While there is no “magic number” of rating scale steps to use, in the tabulated literature the number of steps is generally informed by precedence (e.g., using a rating item previously published) and the number of items to be rated—a large number of items is usually associated with a smaller number of steps per item, as discussed next.

#### Number of emotions rated

Konečni et al. (2008, row 9) deliberately used a single rating item for each stimulus/locus combination, arguing that a large number of rating items might be unrealistic to recall and impractical to complete in response to an excerpt of music (in accord with Viswanathan et al., 2004). Typically 4–6 emotion item ratings were requested per piece, per locus across the tabulated studies. In the open-ended responses, Van Zijl and Sloboda (2011, row 19) asked performers explicitly about emotions they felt when preparing a piece and the emotions that the music was expressing, meaning that there was no explicit limit to the number of emotions that could be reported. Two of the survey studies (Juslin and Laukka, 2004; Zentner et al., 2008, rows 3 and 12) requested participants to rate how frequently a word from a list of emotion terms was appropriate to describe music, on a four step rating item ranging from never to always. The checklist approach pioneered by Hevner (1936, 1937), which lists a relatively large number of emotion words from which the participant can select, is completely absent in the experimental studies tabulated, suggesting utility and ease of statistical analysis of the highly prevalent rating items.

#### Unipolar vs. bipolar rating items

In the experimental studies, response items were most frequently presented in a unipolar format (9 studies: rows 1, 2, 8, 11, 13, 14, 15, 16, and 18), whereas bipolar labels were used for rating items in six studies (rows 4, 5, 6, 9, 13, and 15). Dependent variables were generated from combinations of the rated items in 9 studies, of which 4 were unipolar (rows 1, 2, 11, and 16) and 4 were bipolar (rows 4, 9, 15, and 18), with one study generating an angular variable from the combination of arousal and valence ratings (row 8). Some researchers converted unipolar item ratings into bipolar dependent variables, such as Ilie and Thompson (row 18), who took differences between pairs of unipolar item ratings (e.g., “pleasant” and “unpleasant”) to generate the dependent variable score (e.g., “valence”). That study is also interesting from the point of view that it is one of the few to apply a more contemporary model of emotion dimensions to the data, with energy-arousal and tension-arousal scores generated, instead of the more common “arousal” dimension alone (Schimmack and Rainer, 2002).

The use of bipolar vs. unipolar rating items presents some interesting challenges (Yorke, 2001). A bipolar rating item is labeled at one pole with a term that is opposite in meaning to the other pole to the extent possible (such as “happy” at one end and “sad” at the other). But some researchers have found that supposedly opposite constructs such as happy and sad or excited and calm do not traverse from one pole to the other in a linear, unique, proportionally exclusive manner—that is, they are not exact opposites, do not refer to the identically opposite semantic construct, and do not transition from one to the other in a mutually exclusive manner (it is possible to feel happy and sad at the same time). While space does not permit the discussion of the important question of the distinction between response item formats (see Cacioppo et al., 1997; Larsen et al., 2001; Yorke, 2001), the semantics of rating item labels may interact with interpretations of magnitude when conclusions about locus distinctions are made. This is a crucial matter given that negative emotion rating items (e.g., “Rate how sad” vs. “Rate along a happy-sad bipolar continuum”) and responses to emotions with putative negative emotions (e.g., a piece of *a priori* angry music) are routine design matters in investigations of emotion locus. The increase in experimental efficiency of collecting a single bipolar rating item response (instead of two unipolar rating item responses) weighed against the methodological challenges of using bipolar ratings is an issue that has not been systematically addressed in the emotion locus literature but may have important ramifications.

### Key finding

In nine of the tabulated entries, it was possible to perform a simple count of the number of times the mean internal locus rating was greater than the corresponding mean external locus rating (e.g., rating of internal locus sadness vs. rating of external locus sadness): this is shown as a fraction in the first entry in the Main Findings column of Supplementary Table 1. The counts are based on the cell pairs found in the tables and figures of the publications where direct comparisons of locus pairs were presented. Forty-five out of 178 cell-pair comparisons were higher for internal locus means compared to the external locus mean. If there was no trend, 89 (50%) was the expected count, and so a significantly *small* number of cases had internal locus rated higher [χ^2^_(1, *N* = 178)_ = 43.51, *p* < 0.001]. Furthermore, where significant main effects of locus were reported, external locus ratings were rated higher than corresponding internal locus ratings in eight analyses, and vice-versa in two analyses. On other occasions, difference in mean locus ratings was not significantly different or not reported.

**Figure 1:**
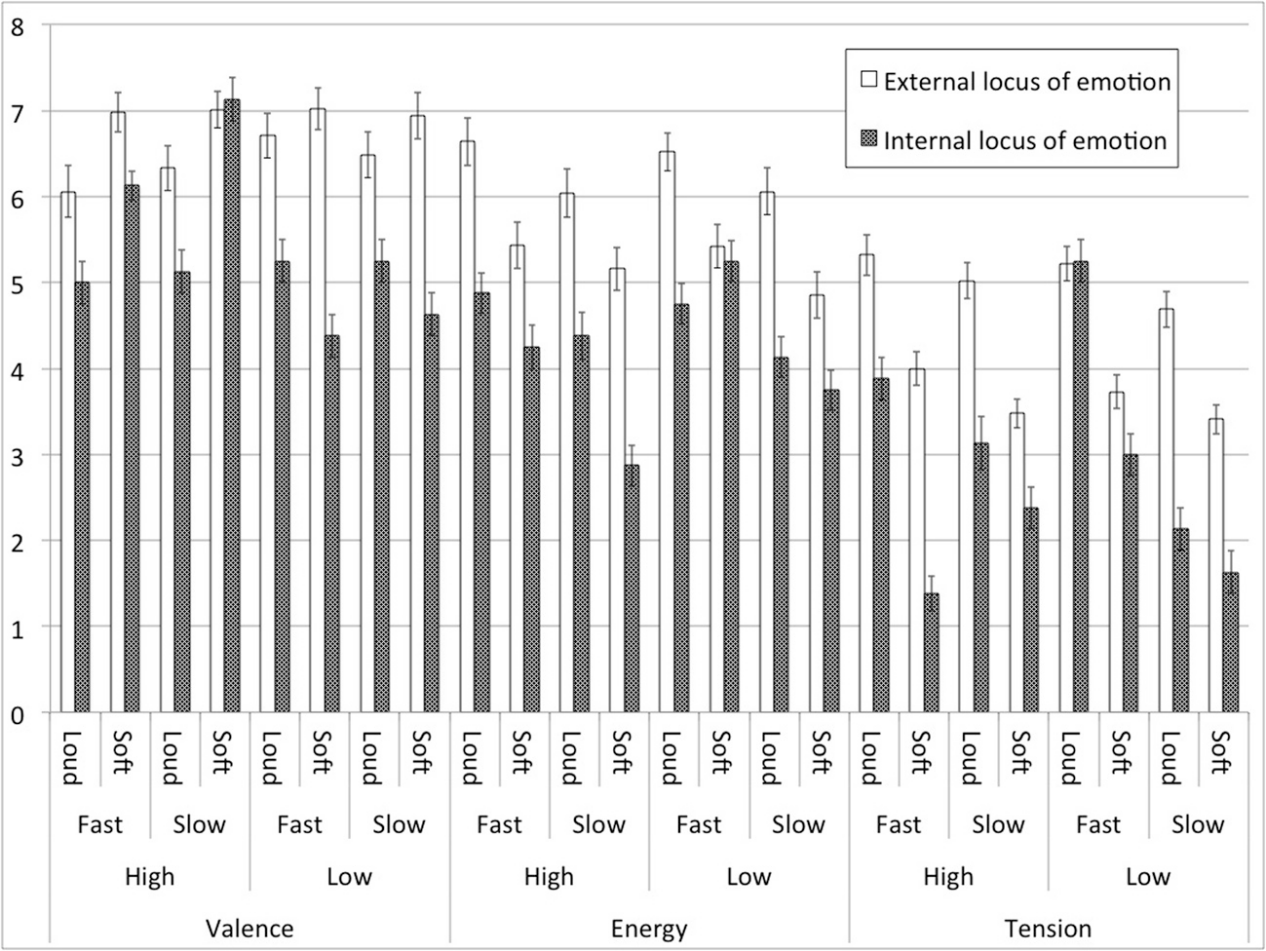
**Collation of data from two experiments (Ilie and Thompson, 2006, 2011, Experiment 1) comparing external and internal locus results across the combinations of independent variables—intensity (loud, soft), rate (fast, slow) and pitch (high, low) for each of three dependent variables (valence, energy arousal and tension arousal) which were formed from combinations of two five-point (0–4) unipolar rating items hence a range of 0–8**. Error bars indicate ±1 *SE*.

The data from the two Ilie and Thompson studies were collated and compared for the current review because those data were designed to allow such comparison. The results reflect the overall findings across the tabulated literature, and so are summarized here. One group of participants (*n* = 27) rated external locus responses in one experiment (Ilie and Thompson, 2006), and another group (*n* = 64) in a separate experiment rated internal locus emotions (Ilie and Thompson, 2011; Experiment 1). The same design and procedure as well as similar stimulus manipulations were used for both experiments with the exception of the musical repertoire used: 5–7 seconds long baroque and classical excerpts for the 2006 study, and a single Mozart piece lasting 7 minutes for the 2011 study. The stimuli were all digitally processed to produce manipulation of pitch, tempo and intensity (two levels for each). The comparisons of mean loci data are summarized in Figure 1. The figure demonstrates the trend found throughout the literature that felt emotions tend to be rated the same or lower than expressed emotions for the corresponding emotion rating and independent variable level combinations.

The use of different stimuli between locus conditions, as is the case in this example, may raise some concerns about the validity of such a comparison, but in that case, one might expect that either a systematic variable has led to the identified trend, or that the number of felt mean scores would be less than the perceived mean score in 50% of pairs (that is, it would be distributed according to chance). As shown in Figure 1, two comparisons have higher felt means than expressed mean ratings: valence rating for the soft, slow, high pitch condition—7.13 vs. 7.01, and tension rating for the loud, fast, low pitch condition—5.25 vs. 5.22. But for each of these pairs, the (1SE) error bars overlap.

While the tabulated literature did not allow a complete, direct statistical comparison of the relative mean magnitude of felt and expressed locus responses, a crude comparison using error bar overlapping was conducted, shown in the magnitude column of Supplementary Table 1 when available or extractable (see note for that column for more details and limitations). A count of these crude “significance” tests revealed that overall there were 99 cases where mean felt emotions were rated as lower in magnitude (regardless of the emotion rated) than the mean expressed rating (for the corresponding emotion rating item), but only nine occasions where the reverse was the case (mean expressed magnitude lower than mean felt magnitude). In 77 tests, error bars between mean locus pairs overlapped, suggesting a relatively high proportion of cases where participants rank emotions as being well matched in magnitude across locus.

In one of the two studies where mean internal locus was rated as higher than mean external locus, it was valence that exhibited a main effect, with mean felt emotions rated as above zero, and mean expressed emotions as below zero (Kallinen and Ravaja, 2006). In the same study, a second main effect was reported where negative activation ratings were also rated in this order across loci. Furthermore, an interaction was identified for the valence score, where pieces that were expected to represent negative emotions (fear and sadness) were rated with higher felt negative emotions than expressed negative emotions (see Main Findings column in Supplementary Table 1, row 4). The second study where mean internal locus was rated as higher than the mean external locus (Vieillard et al., 2008) produced an overall main effect of higher rating for felt than expressed emotion, using the “best label” score approach.

Apart from these two studies, the overwhelming evidence presented in Supplementary Table 1 is that when emotion loci are not statistically the same, emotion felt is rated lower than emotion expressed by the music—for example, higher positive expressed emotion than positive felt emotion (Dibben, 2004; Schubert, 2007b; Konečni et al., 2008; Zentner et al., 2008; Ali and Peynircioǧlu, 2010; Hunter et al., 2010). The study by Salimpoor et al. (2009) provides evidence that when there is a difference in mean locus rating, the difference is constituted as felt emotion being lower, rather than expressed emotion being higher. This evidence is drawn from the comparison of music selection condition results: in the self-selected condition both felt and expressed emotions were rated with a statistically equal mean of about 7 on a pleasure scale of 1 (neutral) to 10 (extremely pleasurable), but in the experimenter music-selected condition, felt emotions ratings dropped to four while expressed emotion ratings remained at around seven.

Across the tabulated literature a number of interacting variables were investigated to see what other influences bear on locus response, including physiological state, personality, age, music-selection responsibility (participant or experimenter), musical features and, as already described above, the putative emotional connotation expected in the music—for example, when the music is expected to represent negative emotion. Some studies reported relationships between interacting variables and the gap across emotion loci (“GAEL,” the difference between the internal and external locus scores, usually reported as an absolute value). With regard to personality differences, participants exhibiting high scores in trait neuroticism-anxiety and on a scale measuring the behavioral inhibition system (“BIS”, the system that specializes in dealing with aversive signals) responded statistically with a larger GAEL score than did those scoring low in neurotic-anxiety and BIS (Kallinen and Ravaja, 2006). Kallinen and Rajava argued that high neurotic-anxiety participants suppressed emotional *experiences* more than their extraverted counterparts (p. 195). In the Salimpoor et al. study, the method of stimulus selection mattered, with participant-selected music producing more equal ratings across loci (smaller GAEL) than experimenter-selected ratings (Salimpoor et al., 2009). Furthermore, the statistically equal ratings between felt and expressed emotion are found when liked music is used, compared to disliked music (Schubert, 2007a, 2010). This is consistent with Salimpoor et al. because the self-selected pieces were presumably liked more than the experimenter-selected pieces in that study.

Hunter et al. (2010) identified interactions between loci and each of the musical feature variable manipulations investigated—tempo and mode—for their specially manipulated stimuli. While they found the overall lower emotion ratings for internal compared to external locus conditions, happiness ratings amplified this difference when the tempo was fast, and sadness ratings amplified this GAEL when tempo was slow (for a summary of other interactions in this study, see Main Findings column of Supplementary Table 1, row 14). Schubert (2007a) reported systematic differences in locus due to preference, with loved music producing a close match between locus scores (small GAEL), a result that was replicated (Schubert, 2010), albeit with fairly marginal significance (*p* = 0.045—see Supplementary Table 1, row 15).

Apart from a main effect of internal locus emotions being rated lower than external locus emotions under many conditions, few of the additional independent variables investigated in the tabulated literature have produced consistent responses, most importantly because of a lack of replication. The most commonly repeated investigated interaction across the tabulated studies is the putative emotion of the musical stimuli. But, as mentioned above, the results are not altogether consistent across studies, and furthermore, there are different ways of collecting emotion ratings (e.g., different ways of labeling of the rating items—see discussion under Response Format, above), transforming those data into dependent variables, and the nature of the musical stimuli.

The clearest, new trend, then, that has emerged in the literature since Gabrielsson's review is that when there is a mismatch between felt and expressed emotion, *and* when these data are gathered via rating items (as distinct from selection of discrete emotions: see Eerola and Vuoskoski, 2011), results rarely show that felt emotions are rated as statistically higher than expressed emotions. As a consequence, the next section of this review focuses on building theory that explains this finding about emotion locus in music.

## Theoretical considerations

### Felt emotion less than expressed: emotional contagion theory

As an extension of Gabrielsson's thinking described in the opening of this paper, it could be assumed that there is no differentiation between the conceptualization of internal and external loci of emotion: it is all part of the same emotional response, and being asked to make the distinction may even be considered artificial. But the findings of the literature reviewed, and in particular the explicit investigation by Juslin and Laukka (2004), demonstrate that a majority of people can make distinctions between these perspectives, understanding that music can appear to express an emotion and that the emotion can be felt. External and internal loci do not necessarily meld into the one experience, at least not for the wide majority of the participants in the Juslin and Laukka study. Even if this distinction is a purely artificial or cultural one, it appears to be widely present and is in need of theoretical understanding.

However, the reason for the quantitatively different rating between loci found in the tabulated literature, when it occurs, could be something as basic as the instructions. Konečni (2008) demonstrated that the wording and detail of the task can impose a secondary influence on the results. He revealed different amounts of detail about a publication on music and emotion and asked the participants to determine which locus the paper was referring to at the different stages. The locus indicated in the article was external and revealed in the title (“… perceived intensity of emotion”)^5^. In Konečni's study, only 25% of participants identified the correct locus, suggesting they tend to think in terms of their own feelings when there is “ambiguity” in wording. The Konečni study demonstrates the importance of clarity of communication. But a theoretically interesting question is why such bias in interpretation may be present.

To explain locus relationships, Evans and Schubert (2008) drew on the distinction made between absolutism and referentialism in the experience of music, as proposed by Meyer (1956) and reinterpreted by Schubert and McPherson (2006). Referentialism suggests that connections between music, emotions, and other situations/events are made by association primarily as a result of life experiences and cultural knowledge but also through highly individual and even idiosyncratic connections. A mismatch in musical emotion and felt emotion can be explained by these kinds of idiosyncratic, arbitrary pairings, as Gabrielsson (2002) points out. Schubert and McPherson then proposed that meaning can also be encoded more directly into the music (as according to Meyer's account of absolutism, or “absolute-expressionism”), where emotions are directly decoded by the listener [an idea found in the “lens model” proposed by Juslin (1997); Juslin and Lindström (2010)] through an act of mimicry, and neurophysiologically via the mirror neuron mechanism (Schubert, 2007b). An influential, related explanation was proposed by Juslin and Västfjäll (2008), who labeled this kind of process as “emotion contagion.” In other words, emotional contagion is the direct influence upon the listener of the emotion that the music portrays, in the absence of outside “interference” through, for example, idiosyncratic connections—such as the unhappy break-up with a partner when otherwise happy music is playing (“referentialism” according to Evans and Schubert, and “episodic memory” according to Juslin and Västfjäll). Emotional contagion may then be taken as one theoretical position for understanding relationships between emotion loci in music.

Put simply, emotional contagion in music refers to the transmission of an emotion via the auditory sense alone. Bharucha et al. (2006) explain:

Unlike other types of contagions, the germs of emotion transmitted by music seem to require no social interaction—musical emotions are airborne contagions. The social contagion of emotions is thought to stem from the tendency to automatically mimic the social cues of others, such as body posture, movement, facial expressions, and vocal expressions. It is perhaps the latter that leads to social contagion in music. (p. 156)

Several of the reviewed studies (Gabrielsson, 2002; Dibben, 2004; Kallinen and Ravaja, 2006; Ali and Peynircioǧlu, 2010; Schubert, 2010) refer to the possible role of social inhibition and the laboratory setting. Under such circumstances, strong emotional outbursts can be considered inappropriate, leading participants to suppress felt emotional response relative to external locus rating. By adopting an emotional contagion framework, what this means theoretically is that inhibition of experienced emotion can take place when making internal locus responses. Evidence from non-music literature about social inhibition and its influence on felt emotion can be found in the within-internal-locus distinctions identified in social psychology where public displays of emotions do not identically map onto actually felt emotions (Bono and Vey, 2005; Mann, 2005; Bakker and Heuven, 2006; Langer et al., 2007; Manne et al., 2007; see also discussion in Limitation 5, above). However, further investigation will be required to examine whether this kind of social, contextual adjustment of felt emotion occurs in response to music.

If we apply contagion theory to explain emotion locus relationships in music perception, that expressed emotions are transmitted to (or infect) the listener, then we can explain inhibition as a plausible explanation for reduced felt ratings, and the inhibition may be a product of social context. Evidence from a study where internal locus emotions were rated in response to various emotion expressing film excerpts (Jakobs et al., 2001) suggests there is an influence of social context. When a film extract was viewed alone, felt sadness ratings were higher than when viewed with another person (see also Raghunathan and Corfman, 2006; and for a similar design, but using music stimuli, see Liljeström et al., 2012).

### Felt emotion more varied than expressed: decoding theory and the lens model

A further complication of the locus relationship debate is the variability in responses to either locus. If the properties of the artwork—in this case, the relationships among musical features over time—are consistent upon repeated exposures, then it may seem logical to assume that the emotion expressed by that stimulus is also stable, and it is the internal locus that might be more variable, depending, for example, on the mood of the listener/perceiver, as Gabrielsson points out in the “zero correlation” case of his “no-systematic relation” (Gabrielsson, 2002 p. 136). Juslin's lens model, discussed above, can be used to help interpret this situation. If a performer and/or composer encodes particular emotions into a piece of music, the listener's decoding will to some extent be a statistical process, meaning that decoding will not necessarily be the same as the encoded emotion. Felt emotion may be characterized as “encoded emotion plus noise.” Schubert (2007b – see Supplementary Table 1, row 5) tested this explicitly by comparing the variance for each emotion rating pair across loci to assess the “stability” of the loci, arguing that if one locus had a lower variance than the other, it was more stable. Six *F-tests* out of 20 (4 emotions rated × 5 pieces of music) were significant at *p* = 0.05, with felt emotions demonstrating larger variance than expressed emotions, with three of these being in response to one piece, “Jupiter” from *The Planets* by Holst (for ratings of emotional strength, arousal and valence). One out of 20 may have been significant by chance alone, and so the study concluded that expressed emotions are overall more stable than felt. It suggests that when all things are equal, internal locus equals external locus plus noise, a claim that requires more research (Schubert, submitted).

Thus, the theoretical underpinning of emotional contagion has not yet been fully addressed in the current literature of emotion locus for music. Further studies will need to falsify the idea that felt emotion is lower in absolute magnitude than expressed emotion because of inhibited contagion, and whether there is a systematic difference in variance between the two loci. Cases of felt emotion being greater than expressed will need to be better explained from a theoretical stance before the inhibited contagion account can be fully supported. I will examine one further theoretical position that is able to explain some of the results identified in the tabulated studies.

### Mixed responses: dissociation theory

I am not explicitly concerned in this review with research on music expressing conflicting emotions, such as happy and sad emotions, at the same time (Hunter et al., 2008, 2010). But peculiar to the locus debate is when a complex combination of emotion matches and non-matches occur between and within loci, such as music *expressing* fear but the listener reacts with feelings of embarrassment *and* joy. Let us suppose now that the joyful reaction was due to the memory that the music evokes about something quite personal and private (as per the “episodic memory mechanism” proposed by Juslin and Västfjäll, 2008), and that upon realizing the response was possibly inappropriate in the current setting (e.g., the music was heard in a concert setting where the other audience members were quiet and calm), the listener became embarrassed. Thus, three potentially mismatching emotions are at play here—one the fear expressed by the music, and the two felt emotions (embarrassment and joy).

After the work of Charland and Colombetti (Charland, 2005; Colombetti, 2005), I (Schubert, 2012, 2013) proposed a solution to the conundrum of mixed emotions in music by arguing that there are two qualitatively different kinds of “feeling” (to use the term in a way similar to Zentner et al., 2008) experiences: emotion valence and affect valence. Emotion valence is specific to emotional contemplation, without any necessary approach or withdrawal action readiness (Frijda et al., 1989). Affect valence, on the other hand, is concerned with the action/evaluative response qualities, which can generally be thought of as preferences (including enjoyment, liking and attraction, or lack thereof). Affect valence is the outcome of the music-listening activity and therefore, also encompasses the more powerful aesthetic responses to music, such as awe, spirituality and being moved (Kivy, 1990, 1999; Konečni, 2005). In the example, the listener was experiencing the positive *emotion valence* of joy (internal locus) but then had a negative *affect* evaluation of embarrassment. The Van Zijl and Sloboda study (2011, see quote in Supplementary Table 1, row 19, Main Findings) further exemplifies this separation through the felt emotion in response to the utilitarian task of learning the piece (affect valence: frustration, remain calm) and the *emotion* valence experienced in response to the music (e.g., again from the quote in Supplementary Table 1, peaceful, happy).

The separation of affect valence and emotion valence, although at times non-trivial, is proposed as a way of resolving previous confusion in the literature about some “mixed” emotional responses (an additional example is provided in Table 2). Simple preference (liking, loving, hating) is a typical example of affect valence found in the literature.

**Table 2:** **Revision of Gabrielsson's relationships between felt and expressed emotion loci in music**.

**Relationship**	**Example**	**Possible mechanism/theory**	**Notes**
1. Matched relationships		Contagion	Same as Gabrielsson's “positive” relationship.
• positive to positive	The happy music makes me feel happy.	Contagion	
• negative to negative	The sad music makes me feel sad.	Contagion	
2. Unmatched relationships	The music is happy but makes me feel sad.	Episodic memory/Evaluative conditioning/Referentialism	Presented as a broader alternative to Gabrielsson's “negative”/“opposite,” encompassing any non-matching emotion/affect.
• positive to non-positive			
• non-positive to positive			
• negative to non-negative			
• non-negative to negative			
*3. Complex/Mixed*	The music expresses several emotions/I feel several emotions, some different from the music.	Dissociation/several	This *optional* category can be reduced to simultaneously multiple occurrences of matched and/or unmatched relationships.
4. Interesting Subcategories, (classified within category 1, 2 or 3 above)			
a. Felt level magnitude	The music expresses a lot of sadness, but I only feel a little bit sad.	Inhibited contagion/partial Dissociation	Reflects the common finding that could be a result of inhibited or incomplete contagion, and/or partial dissociation.
b. Affect/emotion blend	The music expresses grief, and it makes me feel a sense of sadness but also a sense of awe.	Contagion + Dissociation	Music expresses an emotion (in the example, it is a negative emotion valence), but, in the example, the listener feels sadness (possible matching, and a negative valence emotion), as well as a positive affect valence (awe). This is a subcategory of either matched and/or unmatched relationships. The example suggests both (or Complex)
c. Differential variance across loci	Felt emotion ratings more varied than expressed emotion ratings	Contagion, Decoding theory	Contagion occurs, but decoding of emotion occurs with statistical noise (as suggested by the Lens model), which might be explained, for example, by the mood of the listener, their cultural immersion and their familiarity with the style of the music.

The affect/emotion valence distinction is explained from the cognitive theoretical standpoint of dissociation theory, where when listening to music we are usually in a state where we “switch off” pain circuits^6^, meaning that we can enjoy negative emotions without the unpleasant negative affect valence (Schubert, 1996, 2009–2010). In the example above, the embarrassed (negative *affect* valence) response meant that the individual was not in a dissociated state and could, as a result, not (or no longer) enjoy the music that, in another context, he or she may have liked very much (positive affect valence). Recent classifications of descriptive adjectives have started to separate groups of terms in ways compatible with dissociation theory. For example, Juslin and Laukka (2004) propose that some emotions are more suitable for inducing in the listener, while others are more apt for being expressed by the music. Being *moved*, *amazed*, or *enchanted* are presented as examples of induced (but not expressed) emotions. By revising the way affect qualities are conceptualized, these may be understood as unique to induction (rather than expression) *because* they are affect valences (rather than emotion valences). Being moved might be a result of feeling sad, or happy (emotion valence), or some other experience(s) which led to the affective response of being moved.

My point is that by differentiating (dissociating) between emotion valence and affect valence, affect/emotion blends can be more simply understood than the otherwise complex responses we appear to have to music. “The music makes me sad, and that gives me pleasure” suggests a negative *emotion* valence of sadness, but a positive *affect* valence of pleasure. There is no need to view sadness and pleasure as conflicting. From a theoretical stance, the pleasure indicates that the listener is in a dissociated state, meaning that negative valence affects are inhibited, and so all emotions (negative and positive) can be enjoyed. Zentner et al. (2008) present a statistically determined grouping of music evoked adjectives, producing nine clusters of word groups, two of which—“wonder” and “transcendence”—actually fit well with the affect valence concept (with terms such as “amazed”, “moved”, feeling of “spirituality”), while the other clusters indicate adjectives more representative of emotion valence. However, terms such as “irritated” (part of the “tension” cluster) are more typically concerned with affect valence—being irritated by a piece of music is a reason that the listener might stop listening, rather than experience as an emotion that she or he can contemplate (Schubert, 2013). Dissociation theory may provide a solution to one of the enduring debates on emotion locus and whether there exists a special set of aesthetic or musical emotions that are activated only when an artistic (musical) activity or thought takes place, distinct from utilitarian emotions experienced in everyday life (for further discussion, see Kivy, 1989; Krumhansl, 1997; Pouivet, 2000; Khalfa et al., 2002; Krumhansl, 2002; Scherer, 2005; Silvia and Brown, 2007; Silvia, 2009; Barrett et al., 2010; Peretz, 2010; Perlovsky, 2010; Juslin, 2011; Juslin et al., 2011; Chan et al., 2013; Juslin, 2013).

## Revision of gabrielsson's locus relationships

Gabrielsson's categories of emotion locus relationships have provided an important framework for encouraging direct engagement with and awareness of the question of emotion locus in music. The current review suggests that the categories may be reworked and calibrated to reflect the current distinctions between loci of which the research community is now aware. Table 2 summarizes the revised relationships and reports possible explanatory mechanisms (Juslin, 1997; Juslin and Västfjäll, 2008; Schubert, 2007b, 2009–2010). Reworded are the terms “positive” vs. “negative” (or “opposite”) relationships—now “matched” vs. “unmatched,” respectively to reserve the former terms for the conventional use of emotion and affect valence (positive/negative). “No systematic relationship” has been absorbed into “unmatched” to reflect the non-matching nature of emotion pairs that are neither “opposite” nor “positive” (referred to as contrapositive by Evans and Schubert, 2006), such as sad and excited, not just those that are directly “opposite” on an emotion-space geometry (such as sad and happy).

If we eliminate the no-systematic relationship category in those cases when it is due to instability of responses over time, or those that do not concern emotional relationships between expressed and felt emotions on any occasions (no systematic relationship, and no relationship), we end up with two broad categories: matched vs. unmatched relationships. The omission of the non-systematic and no relationships are justified by two findings (1) when no emotion is reported in both the felt and expressed loci, the categorization becomes irrelevant (see, e.g., Sloboda and Juslin, 2010, p. 83)—the participant may be having formalist, cognitive or no responses to the music, but without emotion, and (2) no emotion in one locus and some emotion, or even none, in another can be subsumed by the unmatched emotion locus relationship.

The proposed scheme attempts to revise Gabrielsson's set of relationships to bring them into line with the current state of research on emotion locus in music. The theoretical organizing principle of the revision is based on our understanding, assumptions, and limitations of the emotional contagion processes, and its interactions with a dissociation mechanism and the Lens model inspired decoding theory, discussed above.

Thus, in the revised format two main categories of relationships are proposed: matched and unmatched, referring to whether the expressed emotion is reflected in the felt emotion. A third main category of relationships is included, which is referred to as complex/mixed relationships to account for the possibility of both matched and/or unmatched relationships occurring at the same time. This category is reducible to matched and/or unmatched relationships occurring multiple times and/or simultaneously, and so the complex/mixed relationship is provided for completeness rather than necessity. Furthermore, this category allows for convenient discussion of the interesting area of research concerning mixed emotions portrayed and evoked by music (Evans and Schubert, 2008; Hunter et al., 2008, 2010; Barrett et al., 2010; Juslin, 2011; Juslin et al., 2011).

Subcategories can be attached to the two main categories based on the valence of the relations (for matched, positive [expressed] to positive [felt] or negative to negative, and for unmatched, positive to negative and vice versa—see Table 2). The key issue here is the position of the boundary delineating matched vs. unmatched emotion pairs. For magnitude comparisons of the same emotion variable (e.g., rating happiness on a 1–10 continuum for felt and expressed emotion), conventional inferential statistical procedures can be used to determine matched (no difference) or unmatched (different) loci. However, for discrete emotion words, or when emotions are plotted on an affect grid (Russell, 1980; Russell et al., 1989b), the analysis can be more involved. For example, in some contexts it might be sufficient to refer to a calm emotion expressed as being matched with a happy emotion felt. The current taxonomy does not explicitly dictate where the boundary between a matched vs. unmatched emotion pair lies—e.g., whether “happy” and “calm” are matched emotions or not. Furthermore, for discrete emotion words the boundary may be fuzzy or ill-defined. Evans and Schubert (2008) developed a criterion using Euclidean distance between valence (x-axis) and arousal (y-axis) (assuming the two variables to be orthogonal as per Russell, 1979, 1980), and selected a (more or less arbitrary, but conservative) angle in the space about which to identify whether a pair of emotions are matched vs. unmatched. After some experimentation, they selected an angle of 45° in the polar coordinate system within which the emotions were classified as matched. A “within same quadrant” (in a two-dimensional emotion space) approach is also a plausible, though less conservative, approach.

If this geometric approach to differentiating matched vs. unmatched emotions continues to be adopted, then it would be interesting to see if this empirically determined boundary angle could be reproduced in such a way as to group qualitatively similar emotions together, such as applying inferential statistical tests to subtended angles (Mardia and Jupp, 2000). In practice, it is unlikely that such boundaries will be fixed and stable given that a single emotion does not necessarily occupy the same location on an emotion-space, because there exists some fluidity of meaning within and across individuals and cultures (Russell et al., 1989a; Schubert, 1999). Furthermore, investigation of locus relationships using discrete emotions is in need of further attention, because nearly all of the tabulated studies used rating items, reflecting an interest in magnitude of emotion rather than the semantic distinctions of Russell's “circumplex” model of emotion.

## Discussion and conclusions

Gabrielsson's rediscovery and systematic organization of the relationships between felt and perceived emotion during a musical experience can be considered significantly responsible for opening up a new topic of music psychology and in regard to emotion research in general because it brought to focus the distinction between the locus of emotion when the reactions are not between two sentient beings, but between one sentient being and a piece of music. This review identified the key issues that have emerged in subsequent years and proposed three cognitive based theoretical frameworks to help focus further research. The theories of emotional contagion, decoding and dissociation were presented.

Emotional contagion explains why we tend to feel the emotion that music is conveying. Through the inhibition of this felt emotion, we feel emotion at a lower magnitude than the corresponding expressed emotion. This “inhibited-contagion” account will need more research to determine why there would then be any situations when felt emotions were rated as stronger (in magnitude) than expressed emotions. According to the inhibited contagion account, these situations should not occur, but they do (albeit relatively infrequently in the studies reviewed). Furthermore, is inhibited-contagion a satisfactory explanation for unmatched emotions across loci when the emotions are discrete (such as happy, sad, angry, calm)? And does the related idea of decoding theory mean that internal locus will have more variability than external locus? Such a view needs to account for the question of whether the judgment of an observed object (or piece of music) is really sensed in a fixed, stable way by the perceiver (for philosophical views on this matter, see Townsend, 1987). That is, contagion theory has the implication that it is possible to objectively “know” the external locus emotions, for example through examination of musical features. However, external locus of emotion must also be simulated by the perceiver—for example, illusions demonstrate that the thought-to-be-observed object is not always isomorphic with the physical stimulus (Coren and Girgus, 1978)—and this is a view that has not been tested in the tabulated literature, even though it is important in philosophical aesthetics and is attracting interest in neuroscientific research (e.g., Sevdalis and Keller, 2009; Novembre et al., 2012).

Dissociation theory attempts to address confusion in simultaneously differential responses to music within locus—such as experiencing high preference for music that makes that listener *feel* sad (both internal-locus responses). This theory allows researchers to understand that different, apparently conflicting feelings can be experienced at the same time, without having to dogmatically attribute them to one or the other locus of emotion (e.g., the music is sad, and I like that). Contagion and dissociation principles may work hand in hand in the perception of music: under normal, real-life, day-to-day circumstances it might be undesirable and even dangerous to acquire emotion solely through contagion transfer (e.g., feeling angry when someone else is angry—experiencing fear might be more adaptive. See Preston and De Waal, 2002, who also provide a neuroscientific explanation of inhibited contagion). That is, the individual experiences “utilitarian” rather than “aesthetic” emotions (Scherer, 2004). The day-to-day circumstance is one where emotion valence and affect valence are not dissociated, allowing adaptive responses (the anger leads to a negative affect valence). In music listening and other aesthetic contexts, dissociation of emotion valence from affect valence allows contagion to operate unhindered by day-to-day, utilitarian circumstances and can be enjoyed. In other words, according to dissociation theory, affect valence is always positive in an aesthetic context. The context is the cause of the dissociation between emotion valence and affect valence.

Preference being higher when internal and external loci are matched also requires further investigation. It could be that preference can be implicitly measured by how well the two loci are matched—that is, the smaller that gap across emotion loci, the greater the preference. But determining the causal chain will be of interest, too—whether the preference causes the locus to be different, whether the difference in locus causes preference to change, or whether some other variables are involved. Another interesting research question is which mechanism can explain this finding. Dissociation theory and contagion theory can explain the preference through the activation (which is pleasurable) of contagion circuits. Contagion circuits are the same or related to mirror circuits (e.g., Koelsch et al., 2006) and to the positive affects of empathy. But the nature of those circuits, as applied to the proposed cognitive theoretical frameworks, is in need of further investigation. For example, no studies have explicitly attempted to examine whether neural pathways for processing felt emotion might be different from the neural pathways for processing expressed emotions (e.g., see Blood and Zatorre, 2001; Peretz et al., 2004; Menon and Levitin, 2005; Brattico et al., 2009). Contagion-circuit theory assumes both loci have shared pathways (Preston and De Waal, 2002), but none of the data reported explicitly aims to test this assertion.

Trait and personality effects, including behavioral inhibition/activation systems (Kallinen and Ravaja, 2006), absorption (Kreutz et al., 2008a; Garrido and Schubert, 2011a; Herbert, 2011), rumination (Garrido and Schubert, 2013; Wilhelm et al., 2013) and so on (e.g., Rentfrow and Gosling, 2003; Rentfrow et al., 2011), may each impact the way individuals differ in their felt and perceived judgments of music, but apart from Kallinen and Ravaja, little attention has been given to the effect of trait upon emotion locus. Psychological, physical and physiological states may also influence emotion loci relationships in music, but only a single study has examined this in the tabulated literature (Dibben, 2004, see Supplementary Table 1, rows 1 and 2).

The wide range of task wordings may be in need of some standardization, with the name of the locus variable producing little agreement across studies and therefore unnecessary confusion (for example, when performing a database search on the topic). While the wording of the levels within the locus variable can be quite flexible, Konečni (2008) demonstrated that the wording and detail of the task can also affect the results. Furthermore, the reader needs to be clear on the grammatical subject of the locus level (the *music* expresses and the *listener* perceives external locus emotion). More consistency in the labeling used to describe the variable in question is urged. In this review, the term “locus of emotion” or “emotion locus” has been adopted.

The present review calls for a firmer theoretical stance to help direct future research. Interestingly, one of the rarely cited, early writers on the locus relationship had a premonition of a useful theoretical framework for understanding emotion locus in music, with Vernon Lee's idea of “sympathetic imitation,” which in contemporary literature resembles emotional contagion—the dominant theoretical framework that provides a basis for explaining a large portion of the results of the literature investigated in this review.

Finally, based on an examination of the literature published in the decade after Gabrielsson's seminal work, some rearrangements and suggestions have been made that may assist future researchers investigating relationships between emotion expressed by music and emotion felt by the listener in response to music. The data examined and newly arising findings were used to rearrange Gabrielsson's categorization. Reflecting the growing interest in explaining why emotion ratings are different across locus in certain situations, the simplifying nomenclature of “matched” vs. “unmatched” emotion pairs across loci were used in this review as the basis of formulating locus relationships. There is much work to be done in understanding locus relationships, and this review, if successful, should soon become an interim report on the state of the art in the locus of emotion in music.

### Conflict of interest statement

The author declares that the research was conducted in the absence of any commercial or financial relationships that could be construed as a potential conflict of interest.

## Footnotes

^1^Music is used in clinical and laboratory settings to induce mood. For a review, see Vastfjall (2001). When music is selected for such mood induction, it is inferred that the music will affect the listener because the music is expressing the corresponding emotion. This is borne out by some of the studies in mood induction used in fields of research such as music therapy. Although there is a substantial literature that documents these effects (e.g., Kenealy, 1988; Thaut and De L'etoile, 1993; Gendolla and Kruesken, 2001; Kreutz et al., 2002; Larcom and Isaacowitz, 2009; Harkness et al., 2010; Dyck et al., 2011), these research outputs are not reviewed here because the participant is not rating the emotion the music expresses, but (when required to) the way the music makes them feel.

^2^This issue and the relatively small number of comparable studies made it difficult to sufficiently fulfill the “Checklist of items to include when reporting a systematic review or meta-analysis” proposed by Moher et al. (2009). Relevant details (mean, *SD* and *N*) for this review are nevertheless tabulated when available.

^3^“At least” is indicated because use of the same labels in subsequent studies with an author who had already used these labels is not, in general, added to the count. Exceptions are made when the author has used substantially different labels (e.g., Dibben, 2004: compare Measure column for her Experiments 1 and 2, rows 1 and 2 of Supplementary Table 1—both of these experiments are added to the count). Furthermore, when the term happy, sad, etc. is used to *exemplify* a label (such has “happy, calm, joy” to illustrate the meaning of the label “positive emotion”), it is not included in this count. The same applies for all summary data presented in this subsection. Where a term was presented in a language other than English, the English term, as reported in the target article, is used for tallying.

^4^Caution is urged in making such a grouping since “fear” and “scary” are importantly different along a dominance dimension (angry is a dominant emotion; fear/scared are submissive). The grouping is justified only in terms of similarity on typical valence-arousal semantic spaces (Russell, 1980).

^5^Reading only the title of the article produced the greatest confusion in participant belief about the target locus of the article. However, this might not be so unreasonable given the ambiguity of the term “perceived”, particularly in the absence of the grammatical subject of the sentence, discussed earlier, and summarized in Table 1.

^6^The term “circuits” is used here in a cognitive psychology, metaphorical context, rather than in a literal neuroscience way.

## Supplementary material

Supplementary Table 1 **Summary of emotion locus in music studies reviewed**.
