# Supplementary material for: Emotion felt by the listener and expressed by the music: literature review and theoretical perspectives
Source: Front Psychol. 2013 Dec 17;4:837. doi: 10.3389/fpsyg.2013.00837 (PMC3865445; doi:10.3389/fpsyg.2013.00837)
Supplement: Supplementary Table 1 — Summary of emotion locus in music studies reviewed. [file Data_Sheet_1.PDF]

Supplementary Table 1. Summary of emotion locus in music studies reviewed (2003-2012).

Study [type] [1]	Locus Distinction[2]	Partici-pants[3]	Music Stimuli[4]	Measure[5]	Other IVs[6]	Magni-tude[7]	Main Findings[8]
1. Dibben (2004), Expt. 1 [e]	emotion felt - emotion ex-pressed (mode)	48; 18-60; > 7 yrs train-ing/playing	4 CPP; ES; 0.66; verified unfamiliar	5-point: nostalgia, love, agitated-excitement, peacefulness, spirituali-ty, triumph, happiness, sadness, anger, anxiety. Reduced to 4 DVs: High Energy, Low Energy, Positive Emotion, Nega-tive Emotion)	Arousal manipula-tion (Exercise, Relaxation); Va-lence of Music (Positive, Nega-tive); Energy of Music (High, Low)	5:5:6*	6/16.
2. Dibben (2004), Expt. 2 [e]	as above	48 (15,33); 20 (18-28); 24 (8,16) did Int, 24 (7,17) did Ext.	4 CPP; ES; 0.66; verified unfamiliar	5-point: happiness, ex-hilaration, tenderness, serenity, yearning, sad-ness, fear, anger, and frustration. Reduced to 4 DVs: High Energy, Low Energy, Positive Emo-tion, Negative Emotion	Arousal manipula-tion (Exercise-Immediate re-sponse, Exercise-delayed response, Control); Valence of Music (Positive, Negative); Energy of Music (High, Low)	14:8:2*	10/24. • No main effect of locus. Significant interaction between energy and locus, F(1, 42) = 6.0, p = .012 “tendency for low-energy emotions to be expressed more than they are felt, and for high-energy emotions to be felt” (p. 102) although follow-up tests found no significant difference; Significant interaction be-tween locus and energy, F(1, 42) = 10.91, p = .002. “Separate analyses reveal a significant contrast for high-energy pieces only (p < .0001): high-energy emotions are expressed (M = 1.88, SD = 0.27) more than they are felt (M = 1.41, SD = 0.42)” (p. 103).
3. Juslin and Laukka (2004) [s]	perception - in-duction; perceive - experience	141 (64,77); 17-74; 72/141 had >1 yr train-ing	General re-sponses about music, not to specif-ic pieces	4-point: 38 emotion words - how often a word could be used to describe emotion ex-pressed by music; 44 words rated for frequen-cy of felt emotion. Addi-tional questions “If you perceive that the music expresses a certain emotion, do you also feel that emotion?” [Matched] and “how common they are when you listen to music” with four options [Common].		-	[Matched]: Always 6%, Often 65%, Seldom 29%, Never 1%; [Common]: Int more common, ranked first 41%, Int and Ext, ranked first 39%; Ext only, ranked first 14%; neither, first ranked 6%.

Study [type] [1]	Locus Distinction[2]	Partici-pants[3]	Music Stimuli[4]	Measure[5]	Other IVs[6]	Magni-tude[7]	Main Findings[8]
4. Kallinen and Ravaja (2006) [e]	Point of view: Perceived emotional quality of the music (emotion perceived) - emotion music arouses in the listener (emotion felt); objective-subjective; type of rating- felt, perceived; aroused in the listener-emotion quality of the music; [see also quote in main text under sub-heading Locus Terminology.]	32 (11, 21); 23 (19-29); 12 had no musical training	12 CPP; ES; 1	5-point: alert/surprised-inactive/sleepy, energetic/peppy-bored/ vegetated,happy/ satisfied-sad/hopeless; relaxed/calm-fearful/ angry. Converted to four DVs: positive affect(b), negative affect(b), valence(b), arousal(b), each ranging from -8 to +8.	Stimuli related: *A priori* basic emotions (joyful, sad, fearful); Personality: Behavioural Activation System score (BAS), Behavioural Inhibition System score (BIS); Impulsive Sensation Seeking score (ImpSS); Neurotic-Anxiety score (N-ANX)	5:-3	4/12. • M(int) = .87 (SD=1.56) > M(ext)=-.32 (SD=1.13), F(1,31) = 27.43, p < .001 for valence rating, • Significant Interaction with *A priori* basic emotions F(2,30) = 25.03, p < .001: "felt emotion was more positive than perceived emotion in connection with negative music (sadness and fear), whereas, in connection with positive music (joy) the opposite was true. […] fearful music was perceived as negative, but felt as positive" (p. 202).<br>• M(int)=1.20 (SD=1.47) < M(ext)=2.25 (SD=1.07), F(1,31) = 19.10, p < .001 for arousal rating. Significant interaction with BIS, F(1,30) = 18.44, p < .001 and with N-ANX, F(1,30) = 11.31, p < .01: "The difference between perceived and felt arousal was higher among high BIS and high N-ANX scorers than among low BIS and low N-ANX scorers" (p. 204).<br>• Significant interaction with BIS, F(1,30) = 8.45, p < .01 for PA (positive activation ratings): "The difference between perceived and felt PA was higher among high BIS than among low BIS scorers" (p. 204).<br>• Significant main effect F(1,31) = 41.80, p < .001 for NA (negative activation ratings) M(int) = -1.57 (SD=1.35) < M(ext)=-.13(SD=1.15). Significant interaction with *A priori* basic emotion F(2,30) = 10.31, p < .001: "especially fearful music elicited lower felt than perceived NA" (p. 205).<br>• Significant main effect F(1,31) = 12.16, p = .001 of *low* NA ratings F(1,31) = 12.16, p = .001 "Felt unactivated pleasant affect was higher than perceived unactivated pleasant affect" (p. 205).

Study [type] [1]	Locus Distinction[2]	Partici-pants[3]	Music Stimuli[4]	Measure[5]	Other IVs[6]	Magni-tude[7]	Main Findings[8]
5. Schubert (2007b), Expts. 1 & 2 [e]	locus: internal-external; felt-music trying to express	Expt 1: 28 (14 did Int first); 43 Mdn (20-78). Expt 2: 18; 20 Mdn (40-91).	5 CPP; ES; 1-1.5	7-point: Strong emotion-no emotion, positive-negative (b); aroused-sleepy (b)	5 pieces, Dimen-sions, Locus response timing (Separated-Expt 1, Together-Expt 2)	13:22:0	5/35. • Expt 1. Main effect F(1, 24)=15.171, p = .001 for emotion rating. 7/15 post-hoc tests significant: “For all arousal and emo-tional-strength ratings, external (expressed) locus responses were either statistically the same or greater than the corre-sponding internal (felt) locus response. For valence, the ex-pressed emotion was more negative than the felt emotion in the three cases when the differences were significant. Interestingly, each of these three pieces expressed and evoked negative va-lence emotions” (p. 353). Expt 2. • No main effect F(1,15) = 2.378, p = .144 for emo-tion/piece, but trends observed (see opening fraction of this entry).
6. Schubert (2007b), Expt. 3 [e]	as above	29; 75 Mdn (40-91); 3 yrs lessons	as above	7-point: strong emotion-no emotion, positive-negative (b); aroused-sleepy (b), dominant-submissive (b)	Age group (Young-Expt 1 & 2, Old-Expt 3)	4:16:0	3/20. • Significant main effect F(1, 8) = 6.688, p = .032). Com-parison with Expts 1 & 2: “internal locus (felt emotion) drops even more in emotional intensity for elder participants, whereas felt valence remains fairly closely connected with the expressed responses for the corresponding pieces” (p. 359).
7. Schubert (2007a) [e]	as above	65 (10,55); (19-91)	as above	as above		[6:14:0]	2/20. Gap across emotion loci predicts reduced preference, standardised beta =-.209, t = –2.634, p = .009 for linear, step-wise regression.
8. Evans and Schubert (2008) [e]	locus: internal - external;	45 (15, 28); 20 (17-28)	3: 1 CPP ES, 1 Anthem ES; 1 Imag-ined PS; all confirmed familiar; all 0.5-1.0	11-point: strong emo-tion-no emotion (b), positive-negative (b); aroused-sleepy (b), dominant-submissive (b). DV: Angle between emotions mapped on emotion space (valence on x-axis, arousal on y-axis)	Mode of presenta-tion (Imagined, Heard); Self-selected, Experi-menter selected	–	61% matched, 34% unmatched, 5% no-emotion. • Significant main effect of locus F(4, 35) = 6.36, p < 0.001. Mode of presen-tation n.s., F(4, 35) = 0.807, p = 0.524.

Study [type] [1]	Locus Distinction[2]	Partici-pants[3]	Music Stimuli[4]	Measure[5]	Other IVs[6]	Magni-tude[7]	Main Findings[8]
9. Konečni et al. (2008) [e]	Expression or Depiction of Emotion by Music - Induction of Emotion by Music; "YOU FELT . ." - "… describe THE MUSIC selection you just heard"	144 (37, 107)	3 CPP; ES; 3	13-point: very happy - very sad (b); DV transformed to 7-point absolute value about continuum midpoint (7)	Locus order (Internal first vs External first) + three other factors	2:0:0	M(ext)=3.14 > M(int)=2.36, F(1, 216)=17.66, p<.01.
10. Konečni (2008) [s]	The Musical Piece Expressed Emotion(s) - Subjects Felt Emotions While Listening	56	Extracts from an article on emotion in music	5-point: judge if an [article] title-abstract excerpt (*apriori* Int) was about expressed emotion, felt emotion, both, neither, undecided		|	[article] voted Ext only by 13 (correctly), Int only by 4, both Int and Ext by 37, neither by 0, and unsure by 2.
11. Vieillard et al. (2008) Expt. 1 [e]	Instruction Condition; "instructions" of emotion recognition versus experience; recognised-experienced	59 (23,36); 23 (18-48); 32 none, 27 at least some musical training. Of these 39 (20 Ext, 19 Int) did comparable locus task	56; 2 film music, others specially composed; 12	10-point: ''gai'' (happy), ''triste'' (sad), ''épeurant'' (scary) or ''apaisant'' (peaceful); DV: "best (of the four) label" (scored 0 or 1)		0:0:1	M(int) = .82-.91 > M(ext) = .76-.84, F(1, 37) = 4.97; p = .032; η^2 = .118, for intended emotion score.
12. Zentner et al. (2008) Study 2 [s]	Emotion modality: felt-perceived; aroused-expressed	262	5 styles: Classical, Jazz, Pop/rock, Latin American, Techno	4-point: never-frequently. How often term was felt-perceived	Style of Music (Classical, Jazz, Pop/Rock, Latin American, Techno)	29:15:6	10/50. • Ext reported more frequently than Int, F(10, 248) = 47.98, p < .001. Interaction with Style of Music, F(40, 942) = 3.42, p < .001 – classical and jazz Int reported more frequently than Ext.

Study [type] [1]	Locus Distinction[2]	Partici-pants[3]	Music Stimuli[4]	Measure[5]	Other IVs[6]	Magni-tude[7]	Main Findings[8]
13. Salimpoor et al. (2009) [e]	felt in response to the musical excerpt - participant believed the composer was intending to convey	32 (15, 17); 22 (18-36); (0-18)	4 PS; any style; total 18 CPP + 8 variety; ES based on OPS + PS	10-point: sad-happy; 2-point: not at all aroused-highly aroused	Self-selected, Experimenter-selected (based on stimuli selected by other participants)	2:2:0	1/4. Ext > Int for Valence (t(235) = 2.46, p<.01), and Arousal (t(235) =11.96, Self-selected n.s. Experimenter-selected, Ext > Int for Valence & Arousal [F(1, 232)=7.51, p<.01; F(1, 232)=27.15, p<.001 respectively).
14. Hunter et al. (2010) [e]	response: felt-perceived; actual emotional responses - listeners' perceptions of emotions; feeling - perceiving rating; feelings - perceptions	49 (11, 38);-;4 (0-18) training/playing	32 CPP; ES; unfamiliar; MIDI	5-point: sad, happy	Tempo (Fast, Slow), Mode (major, minor), Emotion (Happy, Sad)	7:5:0	0/8; Ext > Int, F(1,48)=36.06, p<.0001, η^2 =.43; three significant interactions "There were two three-way interactions: one among emotion, response, and tempo" F(1,48)=19.13 p<.0001, η^2=.28, "and another among emotion, response, and mode" F(1,48)=11.58, p<.005, η^2= .19. "The former indicated that the two-way interaction between emotion and tempo (i.e., higher ratings of happiness for fast-tempo excerpts, higher ratings of sadness for slow tempi) was stronger for perceiving compared to feeling responses. In other words, for happiness, the difference between perceiving and feeling ratings was magnified for excerpts with fast tempi, whereas for sadness, the difference was magnified for excerpts with slow tempi. Similarly, the three-way interaction among emotion, response, and mode indicated that for happiness, the difference between perceiving and feeling ratings was particularly strong for excerpts in major mode, whereas for sadness, the difference was particularly strong for excerpts in minor mode. Considered jointly, these two three-way interactions confirmed that the difference between perceiving and feeling ratings for happiness was strongest for excerpts with two cues to happiness, smallest for excerpts with two cues to sadness, and intermediate for excerpts with conflicting cues. The pattern was simply reversed for sadness ratings: "The difference between perceiving and feeling ratings was strongest for excerpts with two cues to sadness, smallest for excerpts with two cues to happiness, and intermediate for excerpts with conflicting cues" (p. 52).

Study [type] [1]	Locus Distinction[2]	Partici-pants[3]	Music Stimuli[4]	Measure[5]	Other IVs[6]	Magni-tude[7]	Main Findings[8]
15. Schubert (2010) [e]	locus: The music seems to be expressing - The music makes me feel	25 (12,13); 21	28 CPP + any style; ES + OPS + PS	7-point: emotional strength; valence (b); arousal (b); dominance (b). DV: Euclidean distance of valence (x-axis) and arousal (y-axis) scores	Preference category (Loved, Hated)	–	Gap across emotion loci lower for loved than hated by M=.86. F(1,70)=4.214, p=.045.
16. Ali and Peynircioğlu (2010), Expt. 2 [e]	Type of rating: conveyed-elicited	44. Of these 22 did Int, 22 did Ext.	24 of 32 CPP + jazz + film music pool, ES; 0.33 (but extract repeated 5 times, so 2). 8 stimuli for each of 4 target [intended] emotions, ratings made after third consecutive playing	5-point: happy, joyful, cheerful, calm, relaxed, at-ease, sad, lonely, blue, angry, hostile, and scornful. DV: happy, sad, calm, angry — each formed from the triads of 5-point items, respectively	[Intended] Emotion (Happy, Sad, Calm, Angry)	5:0:0*	Main effect M(ext)= 3.41 (SD=.77) > M(int)=3.02 (SD=.83), Wilks' lambda = .16, F(3, 40) = 70.87, p < .05. interaction with [Intended] n.s. Wilks' lambda = .97, F(3, 40) = 0.45,: "Even though the overall intensities were judged to be less when the task was to judge the emotion elicited by the melody, the difference between the positive and negative emotions was similar in both types of rating tasks" (p.179).
17. Ali and Peynircioğlu (2010), Expt. 3 [e]	as above	64	as above, with half selected at random for five consecutive hearings per stimulus (familiar), the other half heard each stimulus once (unfamiliar)	as above	Intended (as above), Familiarity (Familiar, Unfamiliar)	7:1:0*	M(ext)=3.52 (SD=.81) > M(int)=2.90 (SD=.96), Wilks' lambda = .24, F(3, 60) = 63.33, p < .05. Sig interaction with [Intended], Wilks' lambda = .92, F(1, 62) = 5.33, p < .05: negative emotion stimuli (sad, angry) rated lower for Int than positive emotion stimuli (happy, calm), relative to corresponding Ext.

Study [type] [1]	Locus Distinction[2]	Partici-pants[3]	Music Stimuli[4]	Measure[5]	Other IVs[6]	Magni-tude[7]	Main Findings[8]
18. Ilie and Thompson (2006; 2011), Expt. 1 [e]	Felt – perceived; perceptual ap-praisal-induced experiential	Int: 64 (28, 36); 17-32; 2.73 (0-13) formal mu-sic lessons. Ext: 27 (7, 10); 18-27; 3.4 (0-20)	Int: 64; CPP; ES; 0.1. Ext: 8; CPP; ES; 7	5-point (0 not at all, 4 extremely): pleasant, unpleasant, energetic, boring, tense, calm. DV: combination of pairs to produce three 8-point variables: valence, en-ergy arousal and tension arousal	Musical features: Pitch, Tempo, Intensity	21:3:0*	2/24. See main text, and Figure 1.
19. Van Zijl and Sloboda (2011) [o]	Musical emotion - own emotion	8 (4, 4); 21 (19-22); 14 training (11-17)	1 per partici-pant; CPP + 20th century; PS	Interviews and diary logging using Individual Playing Sheets which had spaces to be filled out grouped into “box-es”, among which, one section for Int and an-other for Ext		|	“participants […] either […] experienced more or less the same emotions as were found to be present in the music (e.g., Musi-cal Emotion: ‘happy’; Own Emotion: ‘happy’), or that they expe-rienced an emotion caused by but different from the emotion present in the music (e.g., Musical Emotion: ‘melancholy and sad’; Own Emotion: ‘peaceful, happy’). In the case of emotions related to the practice activity, the emotions seemed to be posi-tive (e.g., ‘Yay – I can play it’), negative (e.g., ‘Frustration!’), or neither (e.g., ‘Remain calm’). It was found that all participants experienced both music-related and practice-related emotions. Moreover, it was found that the experienced own emotion changed as learning proceeded” (p. 206).

Note:
Int = Internal locus of emotion; Ext = External locus of emotion.

[1] Type of study code is in square brackets: e = experimental/quantitative; o = open-ended; s = survey/questionnaire (no musical stimuli played).

[2] Label for locus variable followed by at least one example of how locus levels (Int-Ext) were referred to in the study.

[3] First set of numbers are N (M, F) indicating N participants, with M male participants and F female participants. N-M-F = number of participants who did not register a gender; Second set of numbers indicate age (range); Third set indicates music training time. All times/ages are in years. Single values are means unless otherwise indicated. Mdn=Median. Additional sets of number are provided to explain, for example, when two groups were used to report separated (between-subjects) locus responses.

[4] First number is the overall number of pieces evaluated; CPP - western art music of the common practice period (~1550-1900 AD); “Variety”/”any style” - indicates a range of musical styles - pop, folk, non-western; ES - experimenter selected; PS - participant selected; OPS - other participant selected; Duration is a range or typical value in minutes; Unless otherwise stated (e.g., “MIDI”), sound recordings were used.

[5] This column shows the list of emotion responses that participants completed in each locus, most commonly using rating items. When rating items are used, the number of points (steps) is indicated, with the lowest rating indicating absence for unipolar scales, and lower arousal/more negative pole for bipolar item (e.g., “sad” in a bipolar rating item that has the poles sad and happy). Labels with poles separated by a dash or followed by “(b)” indicate that the rating item is bipolar. In such cases absolute value is used to determine response magnitude (e.g. a rating of -3 has a magnitude of 3). The labels used to convert these rating items into dependent variables are

provided after “DV” when available. Terms in square brackets are for ease of cross-referencing in main findings column.

[6] This column shows independent variables (with variable levels in parentheses) that were also reported along with locus. They are referred to here as independent variables for convenience as they report additional variables with which locus of emotion was compared. This does not mean that they were always subjected to inferential statistical testing to examine interactions between locus and the listed variable. They are intended to give the reader a broad scope of the kinds of variables that have been investigated in emotional response alongside, or interacting with, locus.

[7] When available or extractable, the three numbers A:B:C show the count of the number of times mean external locus magnitude was significantly greater than (A), the same as (B), significantly less than (C) the mean internal (felt) locus. When inferential statistics are not directly reported, standard error bars were inspected visually for overlap (1SE) as a crude, practical estimate of significance (Schenker and Gentleman, 2001; Belia et al., 2005; Cumming and Finch, 2005). When data tables of cell mean and SD are presented, 1SE bar overlaps were examined by converting SD to SE (these instances are marked with an asterisk, *); Em dash (—) indicates no actual or potential statistical data could be identified for the three numbers. Number of counts shown will depend of the number of analyses reported, and not necessarily related to the number of participants. For example, “3:1:0” indicates that in three statistical tests reported, mean external locus was significantly greater than mean internal locus, for one statistical test there was no difference, and in no cases was the mean external locus magnitude statistically lower than internal locus. In this example, four sets of statistical analyses would have been reported from which these locus results could be extracted. For simple counts of mean values, see next column. Some values reported will be biased against statistically non-significant differences because of the general propensity in research publications to omit non-significant results (Hahn et al., 2002; Chan and Altman, 2005; Williamson et al., 2005; Dwan et al., 2008). When it appears that non-significant results were not reported, the central datum is reported as an en dash (-). When the trio is presented in square brackets “[A:B:C]” it indicates that the data are taken from the immediately above study but reported using a different combination of analysis cells.

[8] Key locus related finding. First fraction (when present) denotes number of times mean felt emotion score is greater than mean expressed emotion score (numerator) for each level of factor combinations, out of the total number possible factor combinations (denominator). Total number of cells is dependent on presentation of figures and tables in the source publication. M(int) and M(ext) are mean scores for each locus, and when the expression of these two sets of means are separated by “>” or “<” it indicates that the mean score on the left-hand side is significantly greater than or less than (respectively) the mean score on the right-hand side. Inferential statistic summaries are provided when possible/meaningful. Interactions are explained using verbatim text from the source article where possible. n.s. = reported as ‘not significant’.
